# Effect of the Chinese New Year Public Holiday on the Glycemic Control of T1DM With Intensive Insulin Therapy

**DOI:** 10.3389/fendo.2022.915482

**Published:** 2022-06-28

**Authors:** Keyu Guo, Jianan Ye, Liyin Zhang, Qi Tian, Li Fan, Zhiyi Ding, Qin Zhou, Xia Li, Zhiguang Zhou, Lin Yang

**Affiliations:** National Clinical Research Center for Metabolic Diseases, Key Laboratory of Diabetes Immunology, Ministry of Education, and Department of Metabolism and Endocrinology, The Second Xiangya Hospital of Central South University, Changsha, China

**Keywords:** type 1 diabetes mellitus, glycemic control, flash glucose monitoring, holiday effect, continuous subcutaneous insulin infusion

## Abstract

**Aims:**

There is limited evidence that evaluates the glycemic control of type 1 diabetes mellitus (T1DM) during the Chinese New Year public holiday in China. The Chinese New Year public holiday represents various challenges to glycemic control, especially in T1DM patients, in China. We aimed to assess the effect of the Chinese New Year public holiday on several glucose metrics using flash glucose monitoring (FGM) in patients with T1DM.

**Methods:**

Complete FGM data for 1 week before, 1 week during and 1 week after the Chinese New Year public holiday were available for 71 T1DM patients treated with multiple daily insulin injection (MDI) therapy (n = 51) or continuous subcutaneous insulin infusion (CSII) treatment (n = 20). The mean age of the study participants was 13 (9, 30) years. Of note, 59.2% of the patients (n = 42) were adults, and 40.8% of the patients (n = 29) were minors. The interval between each two adjacent periods was one week. The indicators of mean glucose, glucose variability and time in different glycemic ranges were analyzed.

**Results:**

The Chinese New Year public holiday was associated with an increase in mean blood glucose (8.2 ± 1.9 vs. 8.9± 2.8; P < 0.001) and time above range (TAR) (26.1% ± 18.1% vs. 31.7% ± 23.9%; P < 0.001) but a decrease in time in range (TIR) (65.7% ± 16.8% vs. 59.9% ± 21.1%; P < 0.001) and coefficient of variation (CV) (38.2% ± 8.2% vs. 36.7% ± 7.7%; P =0.037). There was no statistically significant difference in time below range (TBR). The glycemic control deteriorated during the Chinese New Year public holiday in our study population regardless of age. Interestingly, in the CSII group, none of the metrics of glucose control significantly changed during the Chinese New Year public holiday.

**Conclusions:**

These results suggested that less self-management may worsen glycemic control in the short term, indicating a need for more refined management algorithms during the Chinese New Year public holiday for T1DM patients.

## Introduction

A population based study has shown that China has 13,000 newly diagnosed type 1 diabetes mellitus (T1DM) cases every year despite a low incidence rate of 1.01/100,000 person-years from 2010 to 2013 (1). A recent longitudinal study has demonstrated that T1DM incidence increased from 2.72 to 3.60/100,000 person-years in a Chinese population from 2007 to 2017 (2). The aims of T1DM management are to avoid acute diabetic complications and prevent or delay chronic diabetic complications and mortality as well as maintain quality of life (3). Unfortunately, the mean HbA1c across all age groups reported in the cost, coverage and care (3C) study in China remains as high as 8.9%, and most T1DM individuals fail to achieve desired glycemic control (HbA1c less than 7.5%) (4, 5). The optimal management of T1DM patients requires adherence to both lifestyle management and therapeutic regimens (6). The self-management of diabetes is necessary to achieve treatment goals for individuals with T1DM (7). However, previous studies have reported that holidays are a major obstacle to dietary adherence and medication adherence for T1DM patients (8, 9). Few studies have reported that the lack of daily routine of both T1DM and T2DM patients during the holidays negatively influence glycemic control (10–13), but no Chinese data in T1DM patients is available. Therefore, the present study investigated the effect of holidays on the glycemic control of T1DM patients, using the Chinese New Year public holiday in China. During this time, the following multiple holiday‐related factors may influence glycemic control: increased stress; participation in more social gatherings with high-calorie foods and drinks; enjoyment of salty meals and alcoholic beverages; and limited opportunities to engage in physical activity (13). Blood glucose monitoring is an essential part of T1DM management. The blood glucose monitoring results are important for making and adjusting treatment protocols as well as evaluating the effectiveness. Compared to HbA1c, metrics of continuous glucose monitoring provide an integral component of glycemic control in T1DM patients, which can represent the presence of excess glycemic excursions and, consequently, the risk of hyperglycemia or hypoglycemia and glycemic variability (14). With application of continuous glucose monitoring (CGM) systems, dozens of metrics enable estimation of glycemic control in T1DM patients. In the 21st century, the Chinese New Year public holiday begins on New Year’s Eve, the day before the first of the Lunar Calendar, and lasts until the 6th of the first month. To determine the effect of the Chinese New Year public holiday on changes of glycemic control in Chinese T1DM patients, we measured and calculated the individual changes of the glucose metrics using flash glucose monitoring (FGM) before, during and after the Chinese New Year public holiday.

## Methods

A total of 311 patients with T1DM who used the FGM system (Freestyle Libre, Abbott Diabetes Care, Rome, Italy) from a follow-up queue at the Second Xiangya Hospital Central South University (Changsha, Hunan, China) from January 1, 2019 to May 21, 2021 were included. The inclusion criteria were: 1) They met the WHO diagnostic criteria for diabetes in 1999; 2) Insulin dependent treatment from diagnosis; 3) The patients were treated with multiple daily insulin injection (MDI) or continuous subcutaneous insulin infusion (CSII). Exclusion criteria: 1) Other types of CGM equipment are being used; 2) Recent complications include ketoacidosis, acute and chronic infection, surgery, trauma and other stress states; 3) Long term use of glucocorticoids or immunomodulators; 4) Unwilling to wear FGMs equipment or allergic to equipment; 5) Acute and chronic hepatic and renal insufficiency; 6) Combined with other autoimmune diseases, such as abnormal thyroid function. This study was approved by the ethics committee of the Second Xiangya Hospital of Central South University (Approval No.: 2019-198). Among the 311 T1DM patients who used FGM, 147 cases were excluded because they did not use FGM during the 3 periods mentioned above, and 93 cases were excluded due to the lack of sufficient valid data. A total of 71 patients were recruited for analysis. The study was conceived as a retrospective data collection, and all the individuals provided written permission to access their clinical data. This study was performed according to the Declaration of Helsinki guidelines. All patients provided informed consent to participate in the follow-up cohort study, and the protocol was approved by the Research Ethics Committee of Second Xiangya Hospital, Central South University. All patients received multiple daily insulin (MDI) or continuous subcutaneous insulin infusion (CSII) treatment.

Sensor data were uploaded from the Libre-view web-based software to generate ambulatory glucose profiles (AGPs) and interpretive summary reports. In the present study, we analyzed average glucose, glucose, glucose variability (calculated as the coefficient of variation, CV; MAGE, mean amplitude glycemic excursion; MODD, mean of daily differences; CONGA, continuous glucose overlapping net glycemic action; LBGI, low blood glucose index; and HBGI, high blood glucose index), time in range (TIR, 3.9-10.0 mmol/l), time above range (TAR, >10.0 mmol/l) and time below range (TBR, <3.9 mmol/l) across three periods according to the Chinese lunar calendar as follows: a week before the Chinese New Year public holiday (Period 1, P1: December 16–December 22); during the Chinese New Year public holiday (Period 2, P2: December 30–January 6, next year); and a week after the Chinese New Year public holiday (Period 3, P3: January 13–January 19). The interval between each two adjacent periods was one week.

To evaluate the role of age on glycemic control during the study period, we considered two age subgroups as follows: adult patients ≥18 and pediatric patients <18 years. To assess the potential relationship between insulin infusion type and glycemic control, we also divided patients into the following two subgroups: patients who used MDI therapy (MDI group), and patients who received CSII treatment (CSII group).

SPSS 26.0 software (IBM Corporation, Armonk, NY, USA) was used for statistical analysis. Normally distributed measurement data are presented as the mean ± standard deviation, and skewed data after normality testing (Shapiro–Wilk test) are presented as the median and interquartile range (IQR). Categorical variables are presented as percentages. An independent sample t‐test or the Mann–Whitney U‐test were used to compare differences between groups. Comparisons across the three periods of interest were performed by ANOVA for repeated measures and *post-hoc* Bonferroni test. A *P* value < 0.05 was considered statistically significant.

## Results

The present study population consisted of 71 T1DM patients with an approximately equal proportion of males and females. The mean age of the study participants was 13 (9, 30) years. Of note, 59.2% of the patients (n = 42) were adults, and 40.8% of the patients (n = 29) were minors. The mean duration of diabetes was 1.8 (0.6, 3.8) years. Among the 71 T1DM patients, 71.8% of the patients (n = 51) were treated with MDI therapy, and 28.2% of the patients (n = 20) were treated with CSII therapy. The study participants had a mean HbA1c value of 7.3 ± 1.2%. The demographic details and clinical data of the study population are described in **Table 1**.

**Table 1:** Characteristics of study participants (n = 71).

Variables	Percentages or mean ± SD	Median (IQR)
Age (years)		13.0 (9.0,30.0)
Age classes		
<18	42 (59.2%)	
≥18	29 (40.8%)	
Duration of diabetes (years)		1.8 (0.6,3.8)
Gender		
Male	35 (49.3%)	
Female	36 (50.7%)	
BMI	18.9 ± 2.8	
HbA1c (%)	7.3 ± 1.2	
Insulin treatment type		
Multiple daily injections	51 (71.8%)	
Continuous subcutaneous insulin infusion	20 (28.2%)	
Insulin dose (IU/kg/d)	0.66 ± 0.23	

**Figure 1:**
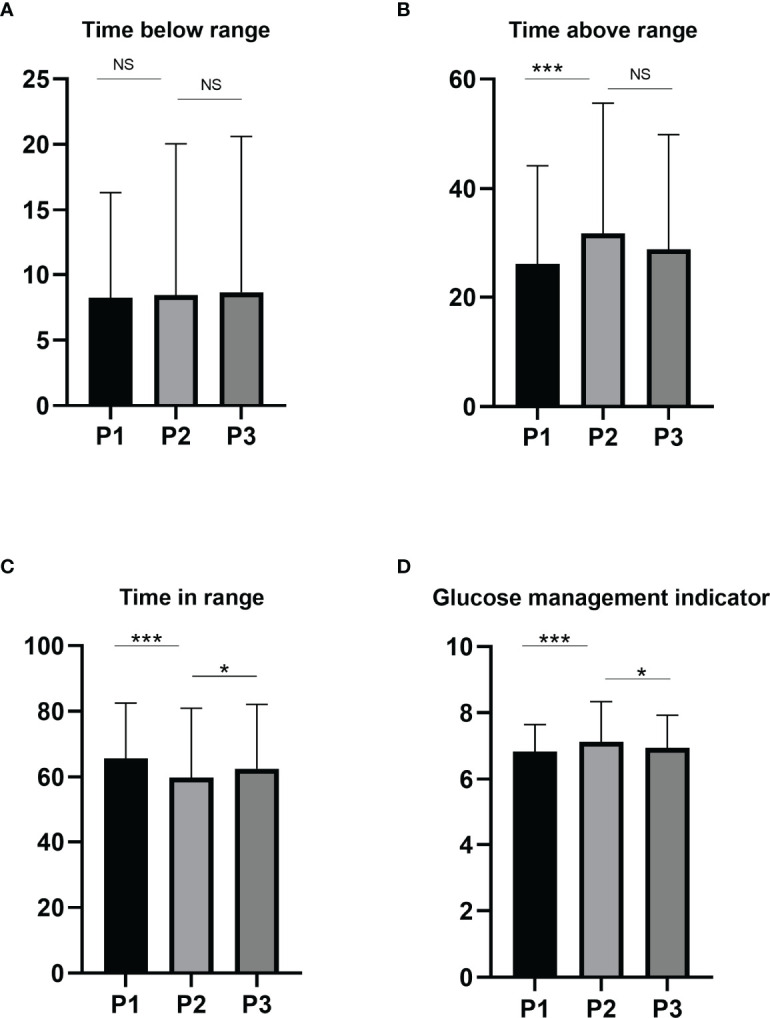
Changes in glycemic indices TBR **(A)**, TIR **(B)**, TAR **(C)** and GMI **(D)** before, during and after the Chinese New Year public holiday. Abbreviations: GMI, glucose management indicator; TIR, time in range; TAR, time above range; TBR, time below range, P1, the period before the Chinese New Year public holiday; P2, the period during the Chinese New Year public holiday; P3, the period after the Chinese New Year public holiday.NS, not significant; *P < 0.05; ***P < 0.001.

**Figure 1** and **Table 2** summarize the main findings during the three periods analyzed. There was a significant reduction of TIR in Period 2 compared to Period 1 and Period 3 (*P* < 0.001 and *P* = 0.014, respectively). Similarly, across the different time points, Period 2 had a significant increase in the TAR (*P* < 0.001 both), HBGI (*P* < 0.001 and p = 0.014, respectively), MBG (*P* < 0.001 and *P* = 0.014, respectively) and GMI (*P* < 0.001 and *P* = 0.015, respectively) compared to Period 1 and Period 3. The TBR remained unchanged between Period 1 and Period 2. Moreover, the CV was not significantly changed between Period 1 and Period 2.

**Table 2:** Changes in CGM metrics of study participants (n = 71) during the Chinese New Year public holiday.

Variables	P1	*P* value^1^	P2	*P* value^2^	P3	*P* value^3^
MBG	8.2 ± 1.9	<0.001	8.9 ± 2.8	0.014	8.4 ± 2.3	0.025
GMI, %	6.8 ± 0.8	<0.001	7.1 ± 1.2	0.014	6.9 ± 1.0	0.025
TBR, %	8.2 ± 8.1	0.803	8.4 ± 11.6	0.787	8.6 ± 12	0.663
TIR, %	65.7 ± 16.8	<0.001	59.9 ± 21.1	0.041	62.6 ± 19.6	0.015
TAR, %	26.1 ± 18.1	<0.001	31.7 ± 23.9	0.051	28.8 ± 21.1	0.026
CV, %	38.2 ± 8.2	0.037	36.7 ± 7.7	0.575	37 ± 7.6	0.078
MAGE	7.3 ± 2.6	0.884	7.3 ± 2.8	0.256	7.2 ± 2.8	0.383
MODD	3.0 ± 1.1	0.062	3.2 ± 1.2	0.014	3.0 ± 1.1	0.649
LBGI	4.8 ± 3.1	0.909	4.7 ± 3.6	0.764	4.9 ± 3.7	0.838
HBGI	8.8 ± 5.5	<0.001	10.6 ± 8.5	0.014	9.4 ± 6.6	0.094
CONGA	7.2 ± 1.8	<0.001	7.9 ± 2.7	0.014	7.5 ± 2.1	0.034

MBG, mean blood glucose; GMI, glucose management indicator; TIR, time in range; TAR, time above range; TBR, time below range; MAGE, mean amplitude glycemic excursion; MODD, mean of daily differences; CONGA, continuous glucose overlapping net glycemic action; LBGI, low blood glucose index; HBGI, high blood glucose index; P1, the period before the Chinese New Year public holiday; P2, the period during the Chinese New Year public holiday; P3, the period after the Chinese New Year public holiday. P value^1^: statistical comparison between P1 and P2. P value^2^: statistical comparison between P2 and P3. P value^3^: statistical comparison between P1 and P3.

**Figure 2:**
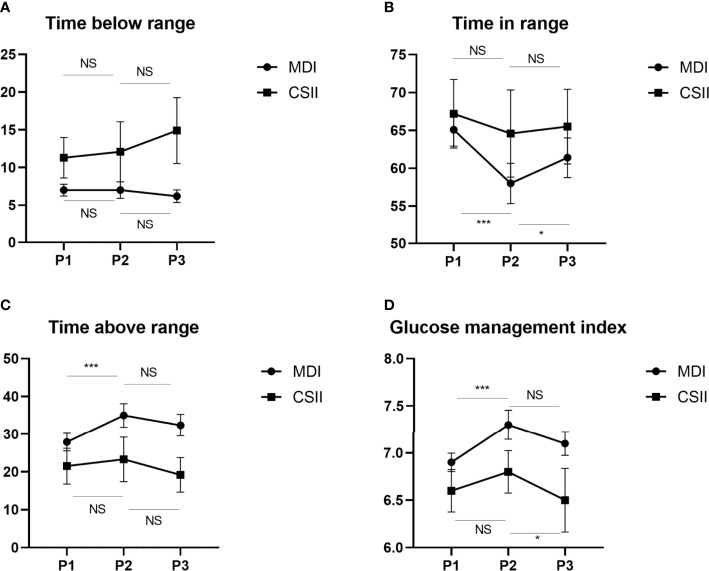
Changes in glycemic indices TBR **(A)**,TIR **(B)**,TAR **(C)** and GMI **(D)** before, during and after the Chinese New Year public holiday in two insulin infusion subgroups. GMI, glucose management indicator; TIR, time in range; TAR, time above range; TBR, time below range, P1, the period before the Chinese New Year public holiday; P2, the period during the Chinese New Year public holiday; P3, the period after the Chinese New Year public holiday. NS, not significant; *P < 0.05; ***P < 0.001.

When considering two insulin infusion types received by the study participants (**Figure 2**), we found that patients in the MDI group had significantly worsened glucose metrics, particularly in terms of the MBG (*P* < 0.001), GMI (*P* < 0.001), TIR (*P* < 0.001) and TAR (*P* < 0.001) in Period 2 compared to Period 1. Better MBG (*P* = 0.02), GMI (*P* = 0.02), TIR (*P* = 0.014) and TAR (*P* = 0.015) values were also observed in Period 3 compared to Period 1. By comparing Period 2 and Period 3, there was little difference in the metrics of glucose control across the different periods in the MDI or CSII groups. Interestingly, in the CSII group, none of the metrics of glucose control significantly changed between Period 1 and Period 2.

**Figure 3:**
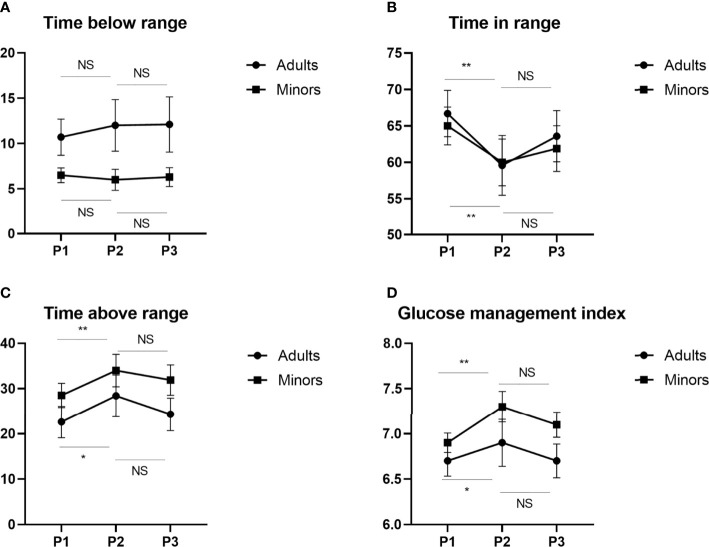
Changes in glycemic indices TBR **(A)**, TIR **(B)**, TAR **(C)** and GMI **(D)** before, during and after the Chinese New Year public holiday in two age subgroups. GMI, glucose management indicator; TIR, time in range; TAR, time above range; TBR, time below range, P1, the period before the Chinese New Year public holiday; P2, the period during the Chinese New Year public holiday; P3, the period after the Chinese New Year public holiday. NS, not significant; *P < 0.05; **P < 0.01.

When dividing the study subjects according to age (**Figure 3**), we found that the TIR in Period 2 was lower than the TIR in Period 1 in both groups (*P* < 0.001 for <18 years, *P* = 0.004 for ≥18 years). The TAR and HBGI was significantly higher in Period 2 compared to Period 1 in both age groups (TAR, *P* = 0.002 for <18 years, *P* = 0.004 for ≥18 years; HBGI, *P* = 0.008 for <18 years, *P* = 0.023 for ≥18 years). Compared to Period 1, an increase in the CONGA value was found both in minor patients and in those aged ≥18 years (*P* = 0.001 and *P* = 0.018, respectively) in Period 2. Com-pared to Period 1, the increase in the MODD occurred only in minor patients in Period 2 (*P* = 0.005), whereas no changes in the MODD were observed in adults. In Period 2, the GMI levels and MBG significantly increased in in both age groups (*P* = 0.001 and *P* = 0.024, respectively) compared to Period 1. However, the TBR levels remained stable across the different periods in both age classes. However, there were no significant differences in the other metrics of glucose control when compar-ing Period 3 to Period 1 or when comparing Period 3 to Period 2.

In order to explore the factors associated with improvement and deterioration of TIR during the Chinese New Year public holiday. We conducted the comparison of clinical characteristics of patients with TIR improved by ≥ 5% vs deteriorated by ≥ 5% between P1 and P2. There was no difference in duration of diabetes, gender, BMI or insulin dose between those with improved vs deteriorated TIR. Our results indicated that there is greater deterioration in TIR for those with higher TIR (77.5% ± 12.1% vs 52.4% ± 13.1%; *P* < 0.001) before the Chinese New Year public holiday. In keeping with this observation, baseline GMI was lower in patients whose TIR deteriorated by 5% or more (6.4% ± 0.5% vs 7.4% ± 0.9%; *P* < 0.001) while baseline HbA1c was not statistically significant (7.0% ± 1.0% vs 7.6% ± 1.5%; *P* =0.078).

## Discussion

In the present study, we aimed to assess the effect of the Chinese New Year public holiday on the glucose profile in T1DM patients by comparing the FGM data in the same cohort before, during and after the Chinese New Year public holiday. During the Chinese New Year public holiday, daily routines were changed, possibly leading to high-calorie diets and reduced physical activity and sleep time. These factors are known to have an impact on glycemic control in T1DM patients. However, there was no obviously difference of any CGMs metrics between the two groups. Thus, we hypothesized that these changes may aggravate glycemic control during the Chinese New Year public holiday.

The main finding of this study confirmed that there was a significant deterioration of almost all the glycemic control metrics as suggested by the increase of TAR values and the reduction of TIR values during the holiday, especially for those with higher TIR and lower GMI before the Chinese New Year public holiday. Although the TBR values remained unchanged, glycemic variability worsened according to the increase of CV. Finally, the mean glucose and GMI levels also increased. Further, the worse glucose profile during the Chinese New Year public holiday appears to be independent of age. However, it can be seen from the **Figure 2** that no matter in which period, the glycemic control of minor T1DM is worse, which indicates that parents need to pay more attention to minor T1DM during the Chinese New Year public holiday and establish a good cooperative relationship between parents and children to jointly manage the blood glucose. A recent study has demonstrated that each 10% reduction in the TIR value is associated with a 36% increase in the risk of diabetic retinopathy and a 60% increase in the risk of microalbuminuria in T1DM patients, indicating that these changes in TIR, TAR and GMI have clinical significance (15). However, the present study only reported the changes in glycemic indices before, during and after the Chinese New Year public holiday in T1DM patients using the FGM system. Continuous glucose monitoring is superior to self-monitoring of blood glucose in improving glycemic control among individuals with T1DM (16, 17).However, the annual cost of FGM for T1DM individuals is 13,372 Chinese Yuan, while the cost of self-monitoring blood glucose levels is only 3675 Chinese Yuan (17). Previous research has shown suboptimal adherence to the treatment regimen and significantly fewer self-monitored blood glucose readings during the holidays in T1DM patients with self-monitoring of blood glucose (12). Because a considerable number of patients cannot afford FGM in China, these patients may have worse control than T1DM individuals with access to FGM during the Chinese New Year public holiday. However, this is only one possibility, and further research on the effects of the Chinese New Year public holiday on T1DM individuals with self-monitoring of blood glucose, which constitute the vast majority of T1DM patients in China, is necessary. The results of our study showed that less self-management may worsen glycemic control in the short term, indicating a need for more refined management algorithms during the Chinese New Year public holiday for T1DM patients. In addition, previous studies have demonstrated an association of weekends and the Chinese New Year public holiday with increased in-hospital mortality in China (18, 19). In addition, there is evidence that Ramadan fasting or COVID-19 lockdown characterized by changes in lifestyle that include sudden changes in mealtimes, sleep, and routine daily activity may affect the glycemic control in T1DM patients from a positive or negative standpoint (20–23). Therefore, strengthening diabetes health education is especially important during the Chinese New Year public holiday to avoid acute and life threating complications, such as ketoacidosis and hyperosmolar coma.

In the present cohort, patients undergoing MDI therapy had a significantly worse glycemic control during the Chinese New Year public holiday compared to those undergoing CSII therapy. However, we also showed that the Chinese New Year public holiday was not associated with worsening or improvement of glycemic control in the subgroup of T1DM patients who were treated with CSII therapy. Previous research has suggested that CSII therapy offers advantages over MDI treatment, such as better glycemic control, reduced hypoglycemia and reduced diabetic ketoacidosis (DKA) events. A recent real-world study has demonstrated that CSII therapy is associated with improved long-term clinical outcomes compared to MDI therapy in China (24). Although the Chinese New Year public holiday introduces various challenges to glycemic control, T1DM individuals treated with CSII therapy did not have worse glycemic control during the Chinese New Year holiday in the present study. Thus, the use of CSII therapy may be a better solution to achieve the desired glycemic control during the Chinese New Year public holiday. However, due to the high cost and patient acceptability of an insulin pump, MDI therapy is often used for the initial therapeutic regimen in T1DM patients in China. To achieve desired glycemic targets in T1DM individuals undergoing MDI therapy during the Chinese New Year public holiday, it is important to enhance the health education of compliance with SMBG, MDI therapy and meals for T1DM patients. Our research team has established a structured T1DM self-management education program entitled, ‘Type 1 Diabetes Education in Lifestyle and Self Adjustment (TELSA)’, which is adapted to medical and cultural practices in China (25). Therefore, the TELSA program and other similar programs may help T1DM individuals to experience a safe traditional Chinese Lunar New Year with better glycemic control.

The present study had a number of limitations that should be noted. First, our retrospective study lacked information on physical activity, sleep status and dietary habits of the study participants before, during and after the Chinese New Year public holiday. In addition, our findings were limited to those who were actively using FGM during the three periods mentioned above and uploaded sensor data in the T1DM clinic, which may impact the generalizability of our findings. Although our subjects were from seven provinces of the South and North of China, most of the patients were from southern China. Considering the differences in climate, eating habits and customs of Spring Festival between the South and North of China, further research needs larger sample size and more detailed subgroup analysis to explore the impact of Chinese New Year public holiday on glycemic control of T1DM in different regions.

## Conclusion

Our results showed that there was worse glycemic control during the Chinese New Year public holiday as suggested by the increase in the TAR value and GMI as well as the reduction in the TIR value. CSII therapy is a better solution to achieve the desired glycemic control for T1DM patients. However, T1DM patients utilizing MDI therapy need enhanced health education to improve their self-management skills to overcome challenges to glycemic control during the Chinese New Year public holiday to achieve optimal glycemic control.

## Data Availability Statement

The raw data supporting the conclusions of this article will be made available by the authors, without undue reservation.

## Ethics Statement

The studies involving human participants were reviewed and approved by the Research Ethics Committee of Second Xiangya Hospital, Central South University. The patients/participants provided their written informed consent to participate in this study.

## Author Contributions

LY and ZZ designed the study, KG conducted the data analyses and drafted the initial manuscript. KG, LZ, JY, ZD, QZ, and QT collected data. All authors reviewed and revised the manuscript, approved the final manuscript as submitted, and agreed to be accountable for all aspects of the work.

## Funding

This work was supported by the National Key R&D Program of China (Grant Nos. 2018YFC2001005) to LY.

## Conflict of Interest

The authors declare that the research was conducted in the absence of any commercial or financial relationships that could be construed as a potential conflict of interest.

## Publisher’s Note

All claims expressed in this article are solely those of the authors and do not necessarily represent those of their affiliated organizations, or those of the publisher, the editors and the reviewers. Any product that may be evaluated in this article, or claim that may be made by its manufacturer, is not guaranteed or endorsed by the publisher.
