# Corrigendum: Effect of the Chinese New Year public holiday on the glycemic control of T1DM with intensive insulin therapy

**DOI:** 10.3389/fendo.2022.985199

**Published:** 2022-07-25

**Authors:** Keyu Guo, Jianan Ye, Liyin Zhang, Qi Tian, Li Fan, Zhiyi Ding, Qin Zhou, Xia Li, Zhiguang Zhou, Lin Yang

**Affiliations:** National Clinical Research Center for Metabolic Diseases, Key Laboratory of Diabetes Immunology, Ministry of Education, and Department of Metabolism and Endocrinology, The Second Xiangya Hospital of Central South University, Changsha, China

**Keywords:** type 1 diabetes mellitus, glycemic control, flash glucose monitoring, holiday effect, continuous subcutaneous insulin infusion

In the published article, there was an error. Due to our mistakes in editing process, the statistical data in the Abstract is not the final correct version, but the data and conclusions in the body text and tables are correct version. A correction has been made to Results section of Abstract.

This sentence previously stated:

“The Chinese New Year public holiday was associated with an increase in mean blood glucose (8.4 ± 1.7 vs. 9.2 ± 2.5; P < 0.001) and time above range (TAR) (27.9% ± 16.6% vs. 35.0% ± 22.3%; P< 0.001) but a decrease in time in range (TIR) (65.1% ± 15.5% vs. 58.0% ± 19.0%; P < 0.001) and coefficient of variation (CV) (65.1% ± 15.5% vs.58.0% ± 19.0%; P < 0.001). There was no significant difference in time below range (TBR)”.

The corrected sentence appears below:

“The Chinese New Year public holiday was associated with an increase in mean blood glucose (8.2 ± 1.9 vs. 8.9± 2.8; *P* < 0.001) and time above range (TAR) (26.1% ± 18.1% vs. 31.7% ± 23.9%; *P*< 0.001) but a decrease in time in range (TIR) (65.7% ± 16.8% vs. 59.9% ± 21.1%; *P* < 0.001) and coefficient of variation (CV) (38.2% ± 8.2% vs. 36.7% ± 7.7%; *P* =0.037). There was no statistically significant difference in time below range (TBR)”.

The authors apologize for this error and state that this does not change the scientific conclusions of the article in any way. The original article has been updated.

## Publisher’s note

All claims expressed in this article are solely those of the authors and do not necessarily represent those of their affiliated organizations, or those of the publisher, the editors and the reviewers. Any product that may be evaluated in this article, or claim that may be made by its manufacturer, is not guaranteed or endorsed by the publisher.

